# Supplementary material for: Research on online book user purchase behavior based on the event logic graph
Source: PLoS One. 2026 Feb 17;21(2):e0341504. doi: 10.1371/journal.pone.0341504 (PMC12912542; doi:10.1371/journal.pone.0341504)
Supplement: S1 File — A detailed statement describing the availability of raw data, computational code, and experimental protocols to support the reproducibility of this study. (DOCX) [file pone.0341504.s001.docx]

Reproducibility Statement

This study adheres to the principles of transparent and reproducible research. To facilitate verification and extension of our findings by other researchers, we provide the following detailed disclosure of data, methods, and computational workflows:

1. Data Source and Collection

Source: Experimental data were collected from publicly available user book reviews on Dangdang.com (a major Chinese online bookstore), covering 119 best-selling titles across 10 vertical categories. The full list of books is available upon request from the corresponding author.

Collection Method: Reviews—including review titles, textual content, and timestamps—were scraped using the legitimate web harvesting tool Octopus Collector (Octopus Data Technology Co., Ltd.).

Time Frame: Data collection occurred between February 27 and March 6, 2025.

Sample Size: The raw dataset comprises 113,499 comments; after preprocessing, 45,017 valid comments were retained for analysis.

2. Data Preprocessing Pipeline

Cleaning Rules: Duplicate entries, comments consisting solely of punctuation or emojis, and texts with ≤5 characters were removed.

Text Processing: Sentence segmentation and word tokenization were performed using the Jieba (v0.42.1) and LTP (Language Technology Platform, v4.1.0) toolkits.

Stopword Filtering: An extended stopword list—including colloquial expressions such as “hahaha” and “hiahia”—was applied to filter non-informative terms.

3. Causal Event Extraction

Rule Templates: Drawing on Chu et al.’s taxonomy of Chinese causal complex sentences (center-linked, end-dependent, and dual-end types), we designed 10 causal pattern templates

Event Representation: Events are represented in a lightweight format combining a core noun phrase and a verb phrase (e.g., “linking theory with practice”).

Automation: Template matching was implemented via regular expressions. The source code is publicly available (see GitHub link in Supporting Information).

4. Topic Clustering and Parameters

Model: Unsupervised topic clustering was conducted using Top2Vec (v1.0.26).

Output: The model generated 16 interpretable topics; representative keywords are listed in Table 3 (e.g., “purchase guidance,” “intellectual resonance”).

5. Event Logic Graph Construction

Tool: Gephi (v0.10.1)

Graph Rules:

Nodes: Represent clustered topics (node size reflects event frequency).

Directed Edges: Indicate causal event pairs (source → target).

Visualization: Layout generated using the Fruchterman–Reingold algorithm, with topic clusters color-coded for clarity.

6. Reproducibility Support

Code & Configuration: Full data processing scripts, causal templates, and Gephi project files are hosted on GitHub under an MIT License.

Data Access: Due to the platform’s user privacy policy, raw review texts cannot be publicly shared. However, the processed structured dataset—including 1,313 extracted causal event pairs—is available from the corresponding author upon reasonable request.

Environment: Python 3.8, TensorFlow 2.7; Top2Vec depends on UMAP-learn 0.5.3.

7. Limitations

The study relies solely on data from Dangdang.com; generalizability across platforms requires further validation.

Researchers may contact the corresponding author (zhangbo@usst.edu.cn or 14248037320@qq.com) to request supplementary materials or consult the detailed implementation guide in the manuscript appendix to reproduce the experiments.

This statement aligns with the FAIR principles (Findable, Accessible, Interoperable, Reusable) and upholds transparency and verifiability standards in computer science and information behavior research.
