# Supplementary figures and images for: Research on online book user purchase behavior based on the event logic graph
Source: PLoS One. 2026 Feb 17;21(2):e0341504. doi: 10.1371/journal.pone.0341504 (PMC12912542; doi:10.1371/journal.pone.0341504)

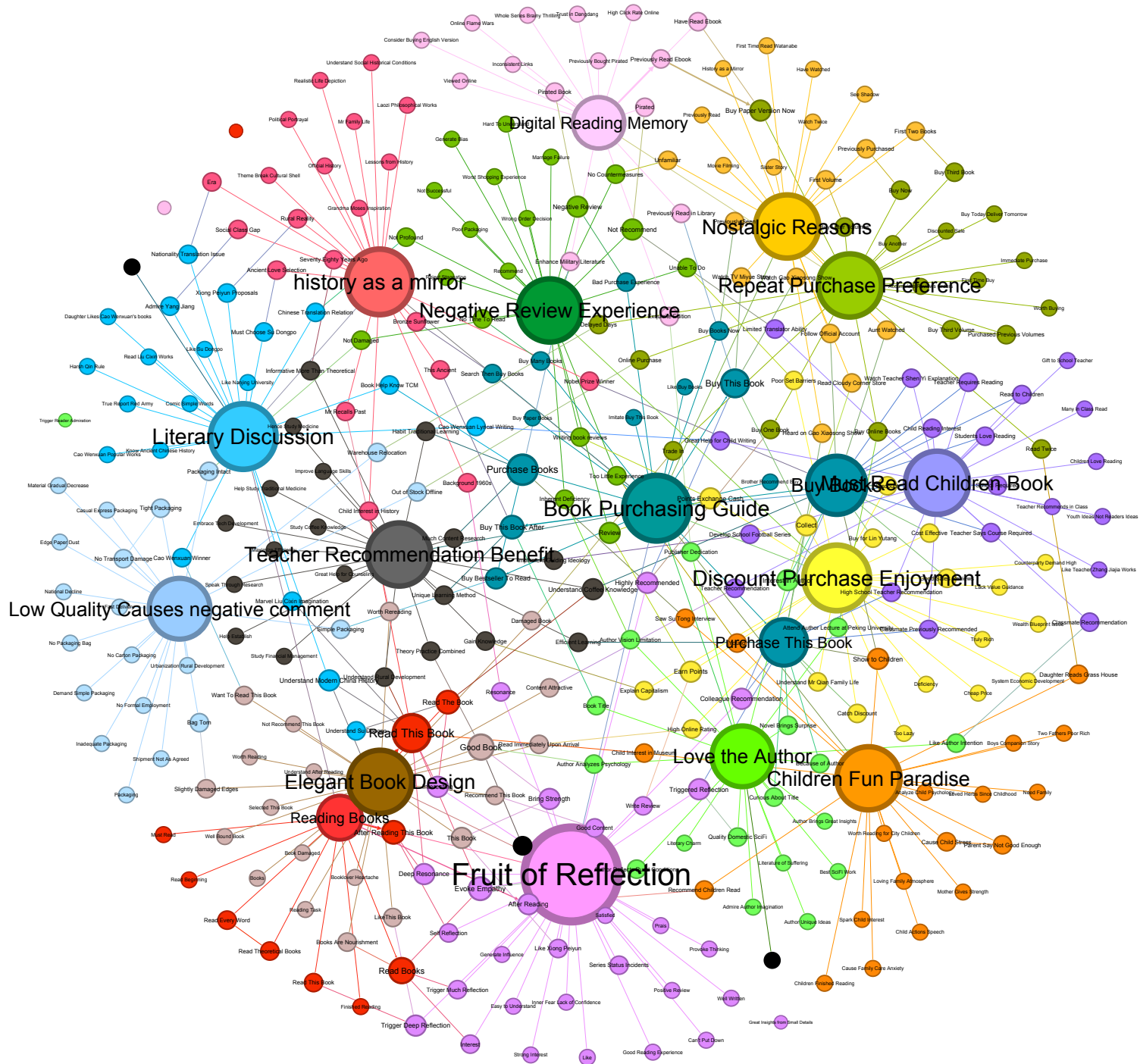

Supplement: S2 File — Supplementary figure showing the full structure of the event logic graph used for modeling online book user purchase behavior, including all observed events and transition paths. (PDF) [file pone.0341504.s002.pdf]
