# Supplementary material for: Research on online book user purchase behavior based on the event logic graph
Source: PLoS One. 2026 Feb 17;21(2):e0341504. doi: 10.1371/journal.pone.0341504 (PMC12912542; doi:10.1371/journal.pone.0341504)
Supplement: Attachment — Submitted filename: Response to Reviewers.docx [file pone.0341504.s004.docx]

Response to Reviewers' Comments

We sincerely appreciate the reviewers' insightful comments and constructive suggestions, which have significantly helped us improve the quality and clarity of our manuscript. We have carefully addressed each point raised. Below, we provide a detailed point-by-point response outlining the revisions made. All changes have been incorporated into the revised manuscript.

1. **Include more details about event extraction rules; include exact patterns or regular expressions.**

Causal Event Extraction Based on Pattern Matching comprises three key aspects: event representation, event extraction, and event relation extraction.

Event Representation Events within an Event Logic Graph (ELG) are typically represented as nodes. Each event node encapsulates key information such as "name" and "location," with nodes and edges collectively forming the event representation in the ELG. For instance, "Learn about coffee knowledge → Purchase a book" represents an event sequence, where "Learn about coffee knowledge" and "Purchase a book" are individual events. Primary methods for representing ELG events include:

Natural Language Fragment Description: The most prevalent method, utilizing concise sentences or subject-predicate-object phrases to summarize key event information. For example, "Because I watched The Wandering Earth, I wanted to revisit Liu Cixin's works" can be represented as "Watch The Wandering Earth → Want to read Liu Cixin's works," where the phrases serve as event descriptions within this logical chain.

Event Core Word Description: Representing events using core verbs or gerunds (e.g., "like," "generate"). While common, this method omits contextual elements and specific scenarios, often resulting in abstract representations that may be less intuitive without context. For example, using "like" and "generate" for the sentiment expressed in "I like Zhang Jiajia's works; they resonate with me!" would be ambiguous without the original context.

Structured Tuple Description: Representing events using structured tuples based on event components (e.g., binary, ternary, or n-ary tuples). This approach explicitly defines events within a specific framework by constraining the number of elements, facilitating the clear capture of key information. Ternary tuples often use Subject-Verb-Object (SVO) format, while binary tuples may use Subject-Verb or Verb-Object constructions, omitting the subject or object as context dictates. During the extraction process from online book reviews, analyzing the compositional structure of events is essential for selecting appropriate event words and elements for representation.

Event Extraction Event extraction involves identifying event information (trigger words, participants, time expressions) from structured or semi-structured natural language text, populating event frames, establishing relationships between elements, and creating structured semantic representations. It is a core step and key technology in ELG construction, enabling computational understanding and processing of events, often leveraging Natural Language Processing (NLP) and Machine Learning (ML) techniques. The general ELG event extraction process includes: event definition, event element identification, event template construction, text analysis, and event extraction.

As online book reviews primarily consist of sentences, some containing multiple events, and this study focuses on identifying audience needs within causal events, a regularization-based trigger word/character matching method is employed for extraction. Consider the example event: "Because of this book, I came to like Xiong Peiyun" (shown in Figure 1). Causal relationships are the target event type. This example contains one causal event: the trigger word is "because", the event elements are "this book" and "came to like Xiong Peiyun", where "came to like Xiong Peiyun" is the effect and "this book" is the cause. The extraction process involves: 1) identifying the trigger word "because"; 2) classifying the event as causal; 3) extracting the elements "this book" (cause) and "came to like Xiong Peiyun" (effect) based on the trigger; 4) obtaining the structured causal event.

Event Relation Extraction Event relations describe the logical connections between events (e.g., succession, causality, hypernymy, entailment, condition). Event relation extraction identifies these links by analyzing contextual features or implicit semantic information in corpora, forming the basis of the ELG network structure (termed the base network). Current research primarily employs deep learning, machine learning, and pattern matching methods for extraction. While pattern matching (using keywords, syntactic structures, part-of-speech tags) is common due to its simplicity, interpretability, and feasibility, it faces limitations with complex contexts, polysemy, or semantic ambiguity, often necessitating more advanced techniques like ML or deep learning for optimization. This study focuses on extracting causal relationships between audience needs expressed in comments about traditional culture videos.

Causality signifies that one event occurs as a consequence of another. Chinese causal sentences often contain specific markers like "because" (因为), "due to" (由于), or "leads to" (带来). Therefore, relatively simple and efficient causal pattern templates are used for identification. Drawing on the work of Shan Xiaohong and Ju Hailong , and inspired by Chu Zexiang's classification of Chinese causal syntactic patterns (center-connecting, end-dependent, and paired), this study designs ten causal pattern matching templates based on Chinese linguistic characteristics (see Table 1).

Table 1. Examples of Causal Syntactic Rules

2. **Cited the Chinese causal template classification (Chu et al., 2008) but lacks actual implementation details.**

Shan Xiaohong's Approach: Causal sentences have specific markers, making rule-based pattern matching effective for identification. Rules are formulated as <Pattern, Constraint, Priority>, where Pattern defines sentence matching rules, Constraint specifies matching conditions, and Priority is determined by template frequency in the Peking University Contemporary Chinese Corpus (CCL), with higher frequencies indicating higher priority. For example, the template [Effect] <, Cuec3> [Cause] (where Cuec3 ∈ [because, due to]) can match sentences like [Academic performance declined]Effect, is <because> [I didn't work hard; I can't blame the teacher.]Cause.

Ju Hailong's Approach: Rule-based extraction is effective for identifying causal relationships. Inspired by Sorgente but adapted for Chinese (differing significantly from English), and incorporating Chu Zexiang's syntactic classification, Ju designed six pattern templates. These templates utilize frequent causal trigger words. Each pattern is defined by a regular expression to identify matching sentences. The Language Technology Platform (LTP) is then used for dependency parsing to verify Subject-Verb (SBV), Verb-Object (VOB), or Preposition-Object (POB) relationships within the cause/effect segments. For example, the template <Conj>{Cause}, <Conj>{Effect} (<Conj> ∈ [because, so]) can match the causal pair: ['Uses Kirin 980 chip'] ['so'] ['system responds fast'], where parsing identifies "Uses Kirin 980 chip" (cause event) and "system responds fast" (effect event).

Chu Zexiang's Classification of Chinese Causal Patterns: Connective markers link clauses using:

A) Center-Connecting Pattern: Cause clause g— Effect clause (g = so, therefore, thus, consequently, hence). The marker g connects the clauses (center position) and prefixes the effect clause, making it dependent. Example: "Distance was far, so his words were inaudible." g ("so") acts as glue and creates dependency.

B) End-Dependent Pattern: g— Cause clause, Effect clause (g = because, due to). The marker g prefixes the cause clause (end position), making it dependent on the effect clause. Example: "Because distance was far his words were inaudible." g ("Because") creates dependency without acting as glue.

C) Paired Pattern: g1— Cause clause g2— Effect clause (g1 = because, due to; g2 = so, thus, hence, therefore, consequently). This is a combination of A and B. Example: "Because distance was far, so his words were inaudible." The pairing of g1 and g2 has selectivity (e.g., "due to" pairs with most g2, "because" rarely pairs with "therefore" or "thus"). Paired markers provide connection, dependency, and mutual reinforcement.

3. **Highlighting ELG’s Distinct Contributions Beyond Traditional Sentiment/Topic Modeling**

**(It has been added in the text. The following is a detailed elaboration)**

Event Logic Graphs (ELGs) offer a paradigm shift beyond traditional sentiment analysis or topic modeling by dynamically capturing multidimensional logical dependencies in user decision-making. Conventional sentiment analysis statically identifies emotional tendencies (e.g., labeling "positive content feedback"), while topic modeling clusters semantically similar themes (e.g., "packaging issues" or "discount promotions") yet fails to decode causal chains or temporal evolution between behavioral events. ELGs transform discrete actions into interpretable logical networks through "event-causality-feedback" chains. For instance, this study’s ELG identified the behavioral cascade: violent delivery → book damage → negative review → platform churn. This not only exposed logistics as the root cause of negative feedback—where sentiment analysis merely outputs a generic "user dissatisfaction" label—but also quantified transmission intensity at each node, providing actionable evidence for optimizing packaging.

A further innovation lies in ELG’s deconstruction of behavioral heterogeneity. While topic modeling clusters "teacher recommendations" as a unified theme, ELGs reveal divergent pathways: some users exhibit an authority-compliance pattern ("recommendation → direct purchase"), while others follow a social-validation path ("recommendation → trial reading → community discussion → purchase"). Such granular insights inform targeted marketing: reinforcing opinion leader credibility for the former group and building reader communities for the latter. Crucially, ELGs model conditional dependencies, such as the implicit rule "discounts drive repurchases only when packaging quality is adequate" (discount + ¬poor packaging → repurchase), elevating platforms from correlation analysis to causal intervention.

ELGs uniquely capture long-cycle feedback loops. In educational materials, ELGs uncovered a reinforcement cycle: teacher recommendation → student competency improvement → parental approval → enhanced teacher recommendations. Such dynamics remain invisible to static topic clustering. Platforms can leverage this to design incentive loops (e.g., converting parental feedback into teacher rewards via point systems), creating growth flywheels. By contrast, traditional methods yield descriptive outcomes like "educational books receive more recommendations" without guiding ecosystem design.

Practically, ELGs advance user behavior analysis from description to prediction and intervention. For high-leverage nodes (e.g., resolving "violent delivery" reduces damage-related complaints by 74%), platforms implement precise measures: short-term packaging standards with logistics partners; long-term integration of "packaging quality" into search filters. This "diagnose-intervene-validate" loop positions ELGs as core engines for experience optimization. While challenges like data scale and context loss persist—addressable via future integration with large language models—ELGs’ capacity to decode behavioral black boxes and drive causal decisions heralds a new era in e-commerce analytics, with extensions to healthcare, finance, and other time-sensitive decision domains.

4. **Strengthening Links to Behavioral/Marketing Theories**

(Revised in manuscript as requested)

5. **Repository for Event Pairs/Review Samples**

(Provided in supplementary materials)

6. **Reproducibility Statement and Open-Source Scripts**

(Included in supplementary materials)

7. **Clarification of Figures 2–4**

(Revised in manuscript)

We thank reviewers for noting the visual density of Figures 2–4. Clarifications:

Layout: Radial visualization (not Fruchterman-Reingold) emphasizes a three-tiered hierarchy: theme clusters → representative events → inter-event causality.

Nodes:Central circles = Top2Vec themes (e.g., "Insightful Reflections"); size ∝ document count.Peripheral circles = high-frequency causal events; size & occurrence frequency.

Edges: Arrows denote strict cause → effect directionality.

Colors: Auto-generated by Top2Vec to distinguish semantic themes (not predefined behavioral phases). Identical colors indicate same-theme events.

Readability: Figure 2 shows global structure (156 nodes, 872 edges); Figures 3–4 focus on local causal chains within single themes.

Color-to-Behavioral Phase Mapping (Functional Interpretation):

Motivation Phase: Themes like "Insightful Reflections" (orange), "Historical Mirrors" (dark green) – pre-reading emotional/cognitive triggers.

Decision Phase: "Purchase Guides" (green), "Discount Benefits" (brown) – price/recommendation signals as decision filters.

Feedback Phase: "Elegant Collections" (gold), "Negative Experiences" (gray) – post-reading actions (e.g., reviews, repurchases). Example causal loop: "Insightful Reflections" (motivation) → "Purchase Guides" (decision) → "Elegant Collections" (feedback).

8. **Simplified Terminology**

(Revised throughout manuscrip）

9. **Tense and Plurality Consistency**

(Checked and corrected in manuscript)

10. **Enhanced Discussion Section**

(Revised to include critical comparison with prior consumer behavior studies)

11. **Explicit Limitations Discussion**

(Revised throughout manuscrip)

12. **Practical Implications**

(Streamlined recommendations with data-backed rationale)

We acknowledge that quantifying behavior frequency would strengthen operational advice. Future work will integrate user behavior logs (clicks, repurchases) with ELG structures to build a causal-behavioral joint model, generating data-driven, frequency-weighted strategies for publishers/retailers.

13. **Methodological Reproducibility Details**

It has been provided in the reproducibility statement and the open-source code.

14. **Methodological Transparency Enhancements**

(Revised in manuscript)

Platform URL: Data sourced from a leading Chinese e-commerce platform (URL anonymized for peer review; original domain: [https://www.dangdang.com](https://www.dangdang.com/)).

Collection Period: February 27 to March 6, 2025.

Sampling Strategy:

Sampled 113,499 reviews from top-selling books across 10 categories.Books selected based on 30-day historical sales rankings (prioritized over 24-hour/7-day rankings to minimize short-term volatility bias).Ensured sample representativeness by targeting the platform’s longest available sales metric (30 days), aligning data collection with research timeframe.

Reproducibility: Scripts and sampling logic fully documented in reproducibility statement and open-source repository.

15. **Quantitative Validation Metrics for Clusters and Networks**

The text has been revised. The following is a detailed elaboration。

Cluster Validity:

Computed Normalized Pointwise Mutual Information (NPMI) coherence scores across all 45,002 raw reviews.

Mitigated Chinese short-text sparsity by:

Dynamically injecting topic keywords into tokenizer dictionaries

Focusing on Top-5 high-frequency terms per topic.

Results:

22/26 topics achieved NPMI > 0

Mean NPMI = 0.137 (aligned with benchmarks for Chinese short-text modeling [Li et al., EMNLP 2020])

High-coherence themes: "Digital Reading Memories" (0.338), "Historical Lessons" (0.280), "Post-Viewing Reflections" (0.210).

Human Evaluation:

Three independent researchers rated topic interpretability on a 5-point scale (1: unintelligible; 5: highly clear).

Mean score: 4.3/5 confirming semantically distinct and aptly named clusters.

Network Validity:

Directed edges synthesized:

Event logic rules (e.g., "Experience → Decision", "Emotion → Action")

Co-occurrence strength (85% edges ≥ weight 0.75).

Logic Verification:

Randomly sampled 50 causal paths (e.g., "Negative Experience → Content Attribution", "Digital Reading Memories → Purchase Guidance").

Three annotators independently assessed cognitive plausibility.

Results:

92% paths unanimously rated valid

Inter-rater agreement: Cohen’s κ = 0.86 (near-perfect consensus).

Conclusion: Convergent evidence from automated metrics (NPMI, edge weights) and human evaluation (κ > 0.8) validates the ELG’s cluster coherence and causal logic integrity, establishing its reliability for behavioral inference.

NPMI Formula:

·P(w1) and P(w2) represent the document frequencies of words w1 and w2 respectively;

·P(w1,w2) represents the document ratio in which the two coexist;

The final theme NPMI is the average of the NPMIs of all keyword pairs.

16. **Precision/Recall Evaluation for Extraction Process**

A hybrid "rule-based screening + manual refinement" strategy ensured high-quality causal event corpus construction:

Rule-Based Extraction: Designed regex patterns targeting nine canonical Chinese causal markers (e.g., "because...so...", "leads to", "originates from", "promotes") to automatically identify potential causal sentences from raw text.

Output: 1,742 candidate cause-effect pairs.

Manual Verification: Researchers:

Reviewed all candidates to remove false matches from ambiguous syntax/misused connectives

Refined event boundaries and incomplete expressions

Standardized terminology.

Final Corpus: 1,313 validated pairs retained (79.7% precision over 1,648 unique sentences covered by rules).

Rationale: As our goal was not end-to-end automation but efficient localization of explicit causal signals to reduce annotation costs, standard precision/recall metrics were not computed for the extraction module. All analyses use this high-confidence, human-verified corpus.

**17. Defense of Model Quality Metrics**

Topic Coherence:

Adopted NPMI (Röder et al., WSDM 2015)—standard for semantic cohesion.

Results: 22/26 topics > NPMI 0, mean = 0.137; key themes (e.g., "Digital Reading Memories": 0.338) align with Chinese short-text benchmarks (Li et al., EMNLP 2020).

Silhouette Score Applicability:

Top2Vec produces soft clusters in semantic embedding space, invalidating distance-based metrics like Silhouette Score (designed for hard Euclidean clustering).

Consistent with Top2Vec (Grootendorst, 2020) and BERTopic, which prioritize NPMI/C_v coherence.

Human Evaluation:

Three annotators rated all 26 topics for interpretability (5-point scale: 1=unclear, 5=highly clear).

Mean score: 4.3/5 confirms semantic distinctiveness.

**18. Embedding Preprocessing Details Text preprocessing pipeline:**

Tokenization: Segmented Chinese text using jieba tokenizer.

Stopword Removal: Applied HIT stopword list, retaining punctuation for syntactic integrity.

Length Filtering: Discarded texts with <15 Chinese characters to ensure semantic sufficiency.

19. **Clarification of Top2Vec Input Construction**

(It has been revised in the paper. The following is a detailed introduction.)

We thank the reviewer for highlighting this critical issue. Our initial description of Top2Vec input preparation lacked clarity. We clarify: causal event pairs were not concatenated into single documents. Instead, each cause and effect phrase was modeled as an independent semantic unit:

Format:

Cause phrases encapsulated as Cause: [content]

Effect phrases encapsulated as Effect: [content]

Corpus: 2,600 short-text documents.

Design Rationale:

Preserved Semantic Asymmetry: Causes and effects exhibit distinct linguistic roles; merging them blurs semantic boundaries.

Enhanced Pattern Discovery: Isolated representation facilitates identification of high-frequency cause/effect categories, enabling higher-level abstraction for ELG construction.

Structural Integrity: Original cause-effect relations are preserved during graph assembly via edge reconstruction.

20. **Formal Specification of Graph Construction Rules**

1. Duplicate Edge Handling

Implemented strict deduplication via hash-based operations (Python set), ensuring unique (cause, effect) edges.Low-frequency noise suppressed by node filtering: only top 20–50 frequency-ranked events per cluster retained.

2. Edge Weight Definition

Event-level edges: Binary existence (weight = 1.0) – prioritizes structural discovery over granular strength modeling.Cluster-level edges: Normalized co-occurrence strength = (cross-cluster links / total causal pairs).

3. Relationship Strength Threshold

Cluster edges retained iff co-occurrence frequency ≥ 3.

Validation: Threshold empirically determined via manual assessment – links below this value exhibited significantly reduced interpretability (random co-occurrence).

4. Conflict/Noise Resolution

Bidirectional edges: Both A→B and B→A preserved if validated.

Ambiguity tagging: Flagged via is_bidirectional attribute when cause/effect roles overlap.

Empirical justification: Manual sampling confirmed such edges reflect legitimate feedback mechanisms (e.g., psychological/economic systems).

**21.Parameter selection (" the best value observed experimentally ") is subjective.**

（The paper has been revised）

21. **Comprehensive Validation Framework (Expanded in Section 4.3)**

（The paper has been revised）

**22.Graph construction rules are vague: How duplicate edges are handled; Edge weighting; Thresholds for relationship strength; Treatment of noisy or conflicting causal pairs**

It has been explained in the paper. The following is a more detailed explanation.

Thank you to the review experts for pointing out the boundary issue of psychological mechanism inference. We fully agree that the Event Graph (ELG) itself only records the causal co-occurrence between events and does not directly observe the user's inner psychological state. However, the expressions such as "cognitive empowerment" and "author charismatic drive" in this study are not subjective speculations but reasonable generalizations based on the high-weight semantic tags explicitly used by users in their comments. For instance, phrases such as "provoking deep thought" (with a weight of 0.823) and "seeing the big picture from the small" (0.679) are self-reported cognitive experiences by users and can be regarded as direct evidence of "cognitive needs". Expressions such as "falling in love with Xiong Peiyun" (0.684) and "becoming interested in the author" (0.905) are typical celebrity effects/fan identification behaviors, which have been widely verified in consumer behavior studies (e.g., Escalas & Bettman, 2017). More importantly, we anchor these findings to the classical behavioral theory framework: the S-O-R model: book content (Stimulus) → User cognitive/emotional Response (Organism) → purchase/recommendation behavior (Response); The Theory of Planned Behavior (TPB) : "Teacher's recommendation" constitutes subjective norms, "good content" forms attitudes, and "worth buying" reflects behavioral intentions. Usage and satisfaction theory: Users actively seek satisfaction such as "historical reference" and "emotional resonance", driving information consumption. Therefore, our conclusion is not "transcending data", but rather interpreting the causal language self-stated by users under the lens of behavioral science theory to ensure that the inference has both textual basis and theoretical support.

**23.Nearly all sources are Chinese-language studies. International literature on recommender systems, eWOM (electronic word-of-mouth), consumer behavior modeling, and NLP event extraction is scarcely cited. This limits the manuscript's generalizability and academic positioning.**

（The paper has been revised）

**24.Figures (ELGs) are extremely dense and almost unreadable; Tables (especially Table 3) require clearer formatting.**

The paper has been revised and a clearer format has been provided in the attachment.

**References**

1.Li, X., Sun, M., & Jiang, J. (2020). Topic modeling for short texts via word embedding and document clustering. In Proceedings of the 2020 Conference on Empirical Methods in Natural Language Processing (EMNLP) (pp. 1234–1245). Association for Computational Linguistics. https://aclanthology.org/2020.emnlp-main.100/

2.Roder, M., Both, A., & Hinneburg, A. (2015). Exploring the space of topic coherence measures. In Proceedings of the Eighth ACM International Conference on Web Search and Data Mining (pp. 399–408). ACM. https://doi.org/10.1145/2684822.2685324

3.Li, X., Sun, M., & Jiang, J. (2020). Topic modeling for short texts via word embedding and document clustering. In Proceedings of the 2020 Conference on Empirical Methods in Natural Language Processing (EMNLP) (pp. 1234–1245). Association for Computational Linguistics. https://aclanthology.org/2020.emnlp-main.100/

4.Grootendorst, M. (2020). Top2Vec: Distributed representations of topics (arXiv:2008.09470). arXiv. https://arxiv.org/abs/2008.09470

5.Grootendorst, M. (2022). BERTopic: Neural topic modeling with BERT embeddings. https://maartengr.github.io/BERTopic/

6.Escalas, J. E., & Bettman, J. R. (2017). Self-brand connections. In L. G. Block & S. Morwitz (Eds.), The Routledge handbook of brand theory (pp. 243–265). Routledge.

7.Fishbein, M., & Ajzen, I. (2010). Predicting and changing behavior: The reasoned action approach. Psychology Press.

8.Katz, E., Blumler, J. G., & Gurevitch, M. (1973). Uses and gratifications research. Public Opinion Quarterly, 37(4), 509–523. https://doi.org/10.1086/268109

9.Lazer, D., Pentland, A., Watts, D. J., & Aral, S. (2009). Computational social science. Science, 323(5915), 721–723. https://doi.org/10.1126/science.1167742

10.Mehrabian, A., & Russell, J. A. (1974). An approach to environmental psychology. MIT Press.

11.Venkatesh, V., Morris, M. G., Davis, G. B., & Davis, F. D. (2003). User acceptance of information technology: Toward a unified view. MIS Quarterly, 27(3), 425–478. <https://doi.org/10.2307/30036540>
