# Supplementary material for: Research on online book user purchase behavior based on the event logic graph
Source: PLoS One. 2026 Feb 17;21(2):e0341504. doi: 10.1371/journal.pone.0341504 (PMC12912542; doi:10.1371/journal.pone.0341504)
Supplement: Attachment — Submitted filename: Response_to_Reviewers_auresp_2.docx [file pone.0341504.s005.docx]

Response to Reviewers

**Manuscript ID:** PONE-D-25-49335R1
**Title:** Research on Online Book User Purchase Behavior Based on the Event Logic Graph
**Journal:** PLOS ONE

Dear Dr. Farshid Danesh and Reviewers,

Thank you very much for your thoughtful evaluation of our manuscript and for the opportunity to revise and resubmit. We sincerely appreciate the time and expertise that both reviewers have devoted to providing constructive feedback. In particular, we are grateful to Reviewer #2 for recognizing the substantial improvements made in the previous revision and for offering valuable suggestions to further enhance the clarity and impact of our work.

We have carefully addressed all remaining comments. The primary focus of this minor revision has been to improve the conciseness, readability, and stylistic precision of the Discussion and Implications sections, as recommended by Reviewer #2. Below, we provide a point-by-point response to the reviewers’ comments. All changes have been highlighted in the revised manuscript using “Track Changes” and are also reflected in the clean version submitted alongside.

Responses to Reviewer Comments

Reviewer #1

“All comments have been addressed.”

We thank Reviewer #1 for their positive assessment and confirmation that all prior concerns have been satisfactorily resolved.

Reviewer #2

“The authors have carefully and substantively addressed the major comments raised in the previous round of review... Only minor issues remain, primarily related to further conciseness and stylistic polishing of the discussion and implications sections. These do not affect the validity of the findings or the overall contribution of the manuscript. I therefore recommend acceptance of the manuscript after minor revision.”

We deeply appreciate Reviewer #2’s generous recognition of our methodological transparency, reproducibility efforts, and theoretical grounding. In response to the suggestion for improved conciseness and stylistic refinement, we have undertaken a thorough revision of the Discussion and Implications sections .Specifically:

We have shortened overly long sentences and eliminated redundant phrasing to enhance readability.

We have replaced promotional or absolute language (e.g., “pioneers,” “propels,” “achieves synergistic alignment”) with more precise, academically neutral terms (e.g., “introduces,” “supports,” “facilitates alignment”).

We have streamlined theoretical references, ensuring each is clearly tied to a specific recommendation without overloading individual sentences.

The core contributions, practical recommendations, and theoretical insights remain fully intact—only the expression has been refined for clarity and adherence to PLOS ONE’s plain-language standards.

These edits appear throughout the tracked-changes manuscript (see lines 387–512 in the revised version) and reflect our commitment to presenting rigorous research in an accessible manner.

Additional Compliance Notes

**Data Availability:** As stated in our Data Availability Statement and Reproducibility Declaration, processed data (including 1,313 causal event pairs), code, and Gephi files are available via GitHub under an MIT License. Raw user reviews cannot be shared due to Dangdang.com’s privacy policy, consistent with PLOS ONE’s data policy exceptions for third-party user-generated content.

**Ethical Compliance:** No human subjects were directly involved; all data were publicly available and collected in compliance with platform terms and Chinese cybersecurity regulations.

**Reference List:** We have verified that all cited works are current and none have been retracted.

Once again, we thank the Academic Editor and reviewers for their insightful feedback, which has significantly strengthened the quality and clarity of our manuscript. We believe the revised version now meets all of PLOS ONE’s publication criteria and hope it will be found suitable for acceptance.

Sincerely,
Dr. Shiling
On behalf of all authors
Email:1424803730@qq.com

Affiliation:Publishing College, University of Shanghai for Science and Technology
